# Selective Internal Radiation Therapy (SIRT) for SDH-Deficient GIST Demonstrates Encouraging Durable Response Rates: An International Multicenter Case Series

**DOI:** 10.3390/cancers18162704

**Published:** 2026-08-20

**Authors:** Zachary T. Berman, Peter Hohenberger, Ramesh Bulusu, Steven C. Rose, Paul T. Fanta, Jonathan Evans, Steffen Diehl, Franka Menge, Nasim Ali, Jason K. Sicklick

**Affiliations:** 1 Department of Radiology, Division of Interventional Radiology, University of California San Diego, San Diego, CA 92103, USA; scrose@health.ucsd.edu (S.C.R.); jsicklick@health.ucsd.edu (J.K.S.); 2 Moores Cancer Center, University of California San Diego, La Jolla, CA 92037, USA; pfanta@health.ucsd.edu; 3 Department of Surgery, University Medical Center Mannheim, Medical Faculty Mannheim, University of Heidelberg, 68135 Mannheim, Germany; phohenberger@posteo.de (P.H.); franka.menge@umm.de (F.M.); 4 Department of Oncology, Cambridge University Hospitals NHS Foundation Trust, Cambridge Biomedical Campus, Hills Road, Cambridge CB2 0QQ, UK; ramesh.bulusu@nhs.net; 5 Department of Medicine, Division of Medical Oncology, University of California San Diego, San Diego, CA 92093, USA; 6 Department of Radiology, Royal Liverpool University Hospital, Prescot St., Liverpool L7 8XP, UK; jonathan.evans@liverpoolft.nhs.uk; 7 Institute of Clinical Radiology and Nuclear Medicine, University Medical Center Mannheim, Heidelberg University, 68167 Mannheim, Germany; steffen.diehl@umm.de; 8 The Clatterbridge Cancer Centre NHS Foundation Trust, Wirral CH63 4JY, UK; nasimali@nhs.net; 9 Department of Surgery, Division of Surgical Oncology, University of California San Diego, San Diego, CA 92093, USA

**Keywords:** SDH-deficient GIST, gastrointestinal stromal tumor, selective internal radiation therapy, radioembolization, yttrium-90, liver metastases, mRECIST, case series

## Abstract

**Background/Objectives:** Succinate dehydrogenase (SDH)-deficient gastrointestinal stromal tumors (GISTs) are a rare subgroup of GISTs and respond poorly to conventional systemic therapies. This study describes long-term outcomes after yttrium-90 (Y-90) selective internal radiation therapy (SIRT) for progression of unresectable SDH-deficient GIST hepatic metastases. **Methods:** We performed a retrospective review of consecutive patients treated with SIRT at three tertiary referral centers in Europe and the United States. Data collection included demographics, tumor profiling, prior therapies, Y-90 dosimetry approach, imaging response, adverse events, and long-term outcomes. **Results:** Twelve patients (66.7% female) with a median age of 27 years (range, 17–57 years) were included. One patient had a complete response (8.3%) and seven had partial responses (58.3%) by modified Response Evaluation Criteria in Solid Tumors. Four patients (33.3%) had tumor shrinkage that did not meet partial response criteria. The objective response rate was 66.7%, with a disease control rate of 100%. One grade 3 or higher adverse event was observed (cholecystitis requiring cholecystectomy). At a median follow-up of 32 months (range, 3–77 months), two patients experienced disease progression. Median overall survival was not reached, with one death during follow-up. **Conclusions:** SIRT appears safe and effective for patients with progressive, unresectable SDH-deficient GIST hepatic metastases, with durable responses and limited serious toxicity. These findings suggest that SDH-deficient GIST may be more sensitive to radiation than previously appreciated and that SIRT may be a useful liver-directed approach for patients with limited systemic options.

## Simple Summary

Succinate dehydrogenase (SDH)-deficient gastrointestinal stromal tumor (GIST) is a rare disease that often affects younger patients and has limited response to standard systemic therapies. Liver metastases are usually unresectable as they tend to be multifocal or bilobar. This international multicenter case series describes 12 patients treated with yttrium-90 selective internal radiation therapy for SDH-deficient GIST liver metastases. Treatment achieved disease control in all patients, an objective response in two-thirds, and durable intrahepatic control with limited toxicity. These findings support liver-directed radioembolization as a useful option for carefully selected patients and justify a multicenter registry-based study.

## 1. Introduction

Succinate dehydrogenase (SDH)-deficient gastrointestinal stromal tumors (GISTs) account for approximately 10% of all GISTs and represent the majority of GISTs arising in children, adolescents, and young adults [1]. They lack the activating mutations in KIT or platelet-derived growth factor receptor alpha (PDGFRA) that define conventional GIST and respond poorly to imatinib and other tyrosine kinase inhibitor (TKI) therapy [2]. The clinicopathologic phenotype is also distinct. SDH-deficient tumors arise almost exclusively in the stomach, occur disproportionately in females, and metastasize to regional lymph nodes, a pattern that is unusual in kinase-mutant disease. Some patients have an inherited tumor-predisposition syndrome, including the Carney–Stratakis dyad, while others have the nonhereditary Carney triad [1,2].

SDH is mitochondrial complex II, a component of the tricarboxylic acid cycle. Functional loss of any SDH subunit destabilizes the complex and causes intracellular succinate accumulation, which can induce hypoxia-inducible factor signaling, altered cellular differentiation, and enhanced angiogenesis. SDH deficiency may result from germline or somatic pathogenic variants in SDHA, SDHB, SDHC, or SDHD, or from epigenetic silencing, most commonly involving SDHC [1,2].

Surgical management remains first-line therapy, and newer chemotherapeutic and targeted approaches are under study in clinical trials [2]. The liver, however, is a dominant site of metastatic spread, and hepatic involvement is typically multifocal and bilobar, so complete resection is frequently not feasible. The natural history of SDH-deficient GIST is also distinct. Patients may survive for prolonged periods despite recurrent or metastatic disease, but the course is heterogeneous and some tumors progress slowly over many years, such that repeated resections reduce disease burden without eradicating the underlying process. Management therefore often combines observation, surgery, investigational systemic therapy, and site-directed treatment according to symptoms, disease biology, resectability, and the dominant site of progression [1,2].

Selective internal radiation therapy (SIRT) with yttrium-90 (Y-90) has an established role as salvage therapy for progressive hepatic metastases after standard treatment for colorectal cancer and neuroendocrine tumors, and offers a way to treat multiple tumor deposits throughout the liver without requiring discrete, resectable or ablatable targets. It is less well studied in GIST. The available experience with hepatic arterial therapy in GIST, comprising chemoembolization and, more recently, radioembolization, derives from molecularly heterogeneous cohorts in which SDH-deficient tumors were a small minority [3,4]. Outcomes from predominantly KIT-mutant disease cannot be assumed to apply to SDH-deficient GIST, because the underlying biology, age distribution, systemic-treatment sensitivity, and rate of progression all differ [2,3,4].

Because SIRT exploits preferential arterial tumor supply rather than a specific actionable mutation, it may be applicable to SDH-deficient GIST irrespective of genotype. The primary aim of this study was to describe imaging response, toxicity, and long-term outcomes after Y-90 SIRT in patients with progressive, unresectable hepatic metastases from biopsy-proven SDH-deficient GIST treated at three tertiary referral centers.

## 2. Materials and Methods

A retrospective review was performed of consecutive patients with GIST treated with SIRT at three tertiary referral centers in Europe and the United States. Patients were included if they had a mutation in SDHA, SDHB, or SDHC. The decision to use SIRT was based on multidisciplinary consensus discussion according to institutional protocol. Deidentified data were collected, including sex, age, clinical indication for SIRT, previous therapies, and tumor genetic profiling.

The SIRT procedure was performed in a staged fashion. First, a mapping/planning angiogram was performed under moderate conscious sedation. A femoral artery puncture was made, and 5F diagnostic catheters were advanced into the celiac and/or superior mesenteric arteries to define hepatic arterial anatomy. Microcatheter selection was then performed to identify the arteries supplying the tumors of interest. At-risk extrahepatic branches were embolized when necessary to reduce the risk of off-target deposition at the time of SIRT. A test dose of Tc-99m macroaggregated albumin was injected, and planar imaging and/or single-photon emission CT was performed to confirm tumoral targeting and calculate lung shunt fraction. After 1–3 weeks, patients returned for a repeat angiogram, during which Y-90 microspheres (TheraSphere [glass], Boston Scientific, Marlborough, MA, USA, or SIR-Spheres [resin], Sirtex, Woburn, MA, USA) were infused. The two devices differ in specific activity and particle number, with resin microspheres generally producing a somewhat greater embolic effect than glass microspheres. Device selection was not protocolized and reflected operator experience and preference, institutional practice, and payer coverage. Activity was calculated using the Medical Internal Radiation Dose (MIRD), partition, or modified body surface area (BSA) method. The dosimetry method was selected by the treating physician at the time of the procedure according to device labeling and treatment intent. Because treatments spanned multiple centers and a prolonged time period, exact sphere counts and some device-specific administration details were not consistently recoverable from the historical record. Given the treatment dates included in this series, all resin microsphere treatments used day-of-calibration specific activity. Patients were discharged home after the procedure.

**Figure 1:**
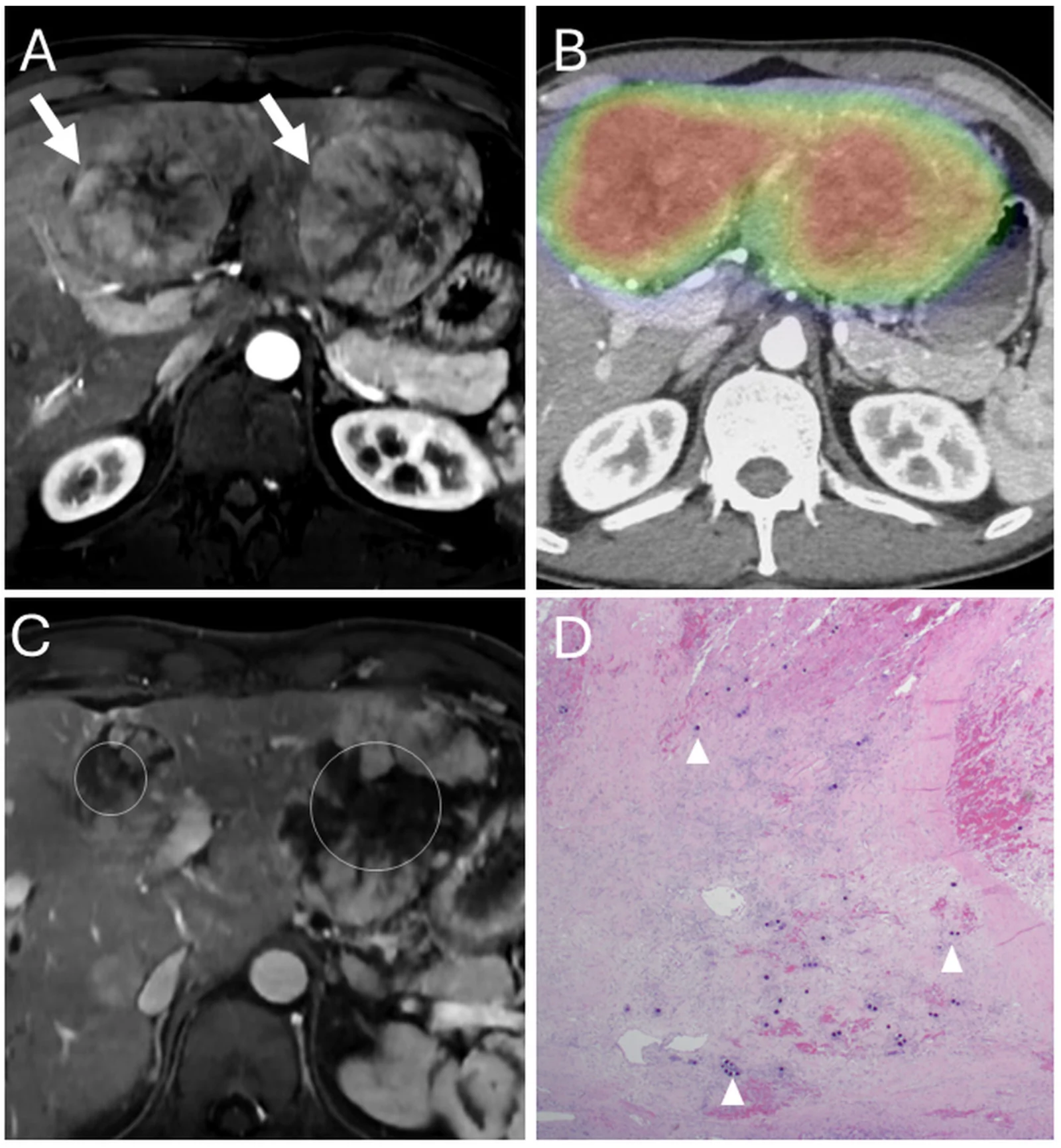
Representative images showing (**A**) pretreatment contrast-enhanced MRI with avid arterial enhancement (arrows); (**B**) fused contrast-enhanced CT with Y-90 SPECT/CT demonstrating radioisotope uptake within left-lobe tumors; (**C**) contrast-enhanced MRI six months after treatment demonstrating increased central necrosis (circles); and (**D**) histopathologic findings from a tumor resected six months after selective internal radiation therapy demonstrating areas of necrosis and microsphere deposition (arrowheads).

Treatment response was assessed on cross-sectional imaging using modified Response Evaluation Criteria in Solid Tumors (mRECIST), which evaluates viable, contrast-enhancing tumor rather than overall lesion size alone, given the propensity of GIST metastases to become necrotic without substantial size reduction after effective locoregional or systemic therapy. Choi criteria were not applied because an enhancement-based assessment was considered more directly relevant after transarterial therapy, for which reduction in viable arterial enhancement may better reflect treatment effect than changes in size or density alone. Surveillance imaging was obtained according to each institution’s standard protocol, generally beginning 4–6 weeks after treatment and continuing at approximately 3-month intervals thereafter. Depth of response was defined as the maximum percentage decrease from baseline in the sum of target-lesion diameters used for mRECIST assessment at any point during follow-up. Progression-free survival (PFS) was calculated from the date of first SIRT to radiographic progression or death, and overall survival (OS) from first SIRT to death; patients without an event were censored at last follow-up. Figure 1 demonstrates a representative case.

Exploratory subgroup comparisons were performed to address treatment heterogeneity. Depth of response was compared using the two-sided Mann–Whitney U test, and objective response rates were compared using Fisher’s exact test.

## 3. Results

A total of 12 patients (8 female, 4 male) were included in this cohort. The median age at the time of SIRT was 27 years (range, 17–57 years). Each had a germline mutation in SDHA (*n* = 6, 50%), SDHB (*n* = 3, 25%), or SDHC (*n* = 3, 25%). Nine patients (75%) had previously undergone partial or total gastrectomy, with or without additional abdominal cytoreduction, before SIRT. Seven patients (58.3%) had received systemic therapy, mainly TKIs, as summarized in Table 1. In exploratory subgroup analysis, patients with prior systemic therapy (*n* = 7) had a median depth of response of 65% compared with 30% among treatment-naive patients (*n* = 5), a difference that was not statistically significant (*p* = 0.22). Objective response rates were 85.7% (6/7) and 60.0% (3/5), respectively (*p* = 0.52).

Given the degree of bilobar involvement, nine patients (75%) received bilobar treatment. One patient had unilobar treatment, one had segmental treatment, and one had a lobar infusion followed by a segmental infusion 2.5 years after the first treatment. Five patients (41.7%) were treated with glass microspheres, six (50%) with resin microspheres, and one (8.3%) received both devices across separate treatments. Among patients treated exclusively with a single microsphere type, median depth of response was 50% with glass (*n* = 5) and 52.5% with resin (*n* = 6), with no statistically significant difference (*p* = 0.71). Objective response rates were 60.0% (3/5) and 83.3% (5/6), respectively (*p* = 0.55); the patient treated with both devices was excluded from these device-specific comparisons. The procedures were generally well tolerated, with one CTCAE v5 grade 3 or higher toxicity. This patient developed cholecystitis after a right-lobar SIRT delivery and required cholecystectomy. The cystic artery was not prophylactically embolized per the institution’s standards. No clinically recognized delayed hepatic toxicity (i.e., radioembolization-induced chronic hepatotoxicity) was documented.

**Figure 2:**
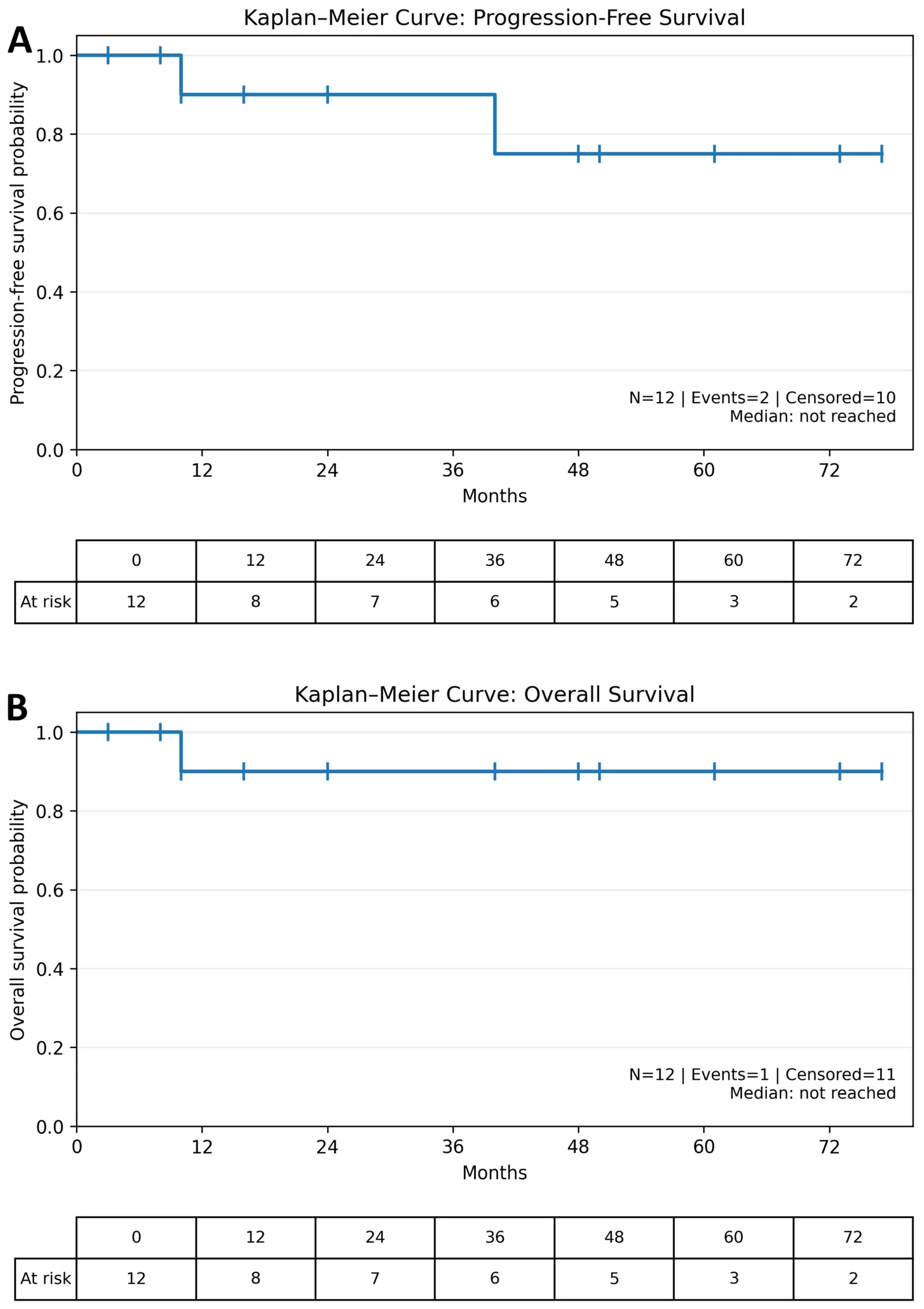
Kaplan–Meier curves of progression-free survival (**A**) and overall survival (**B**).

**Figure 3:**
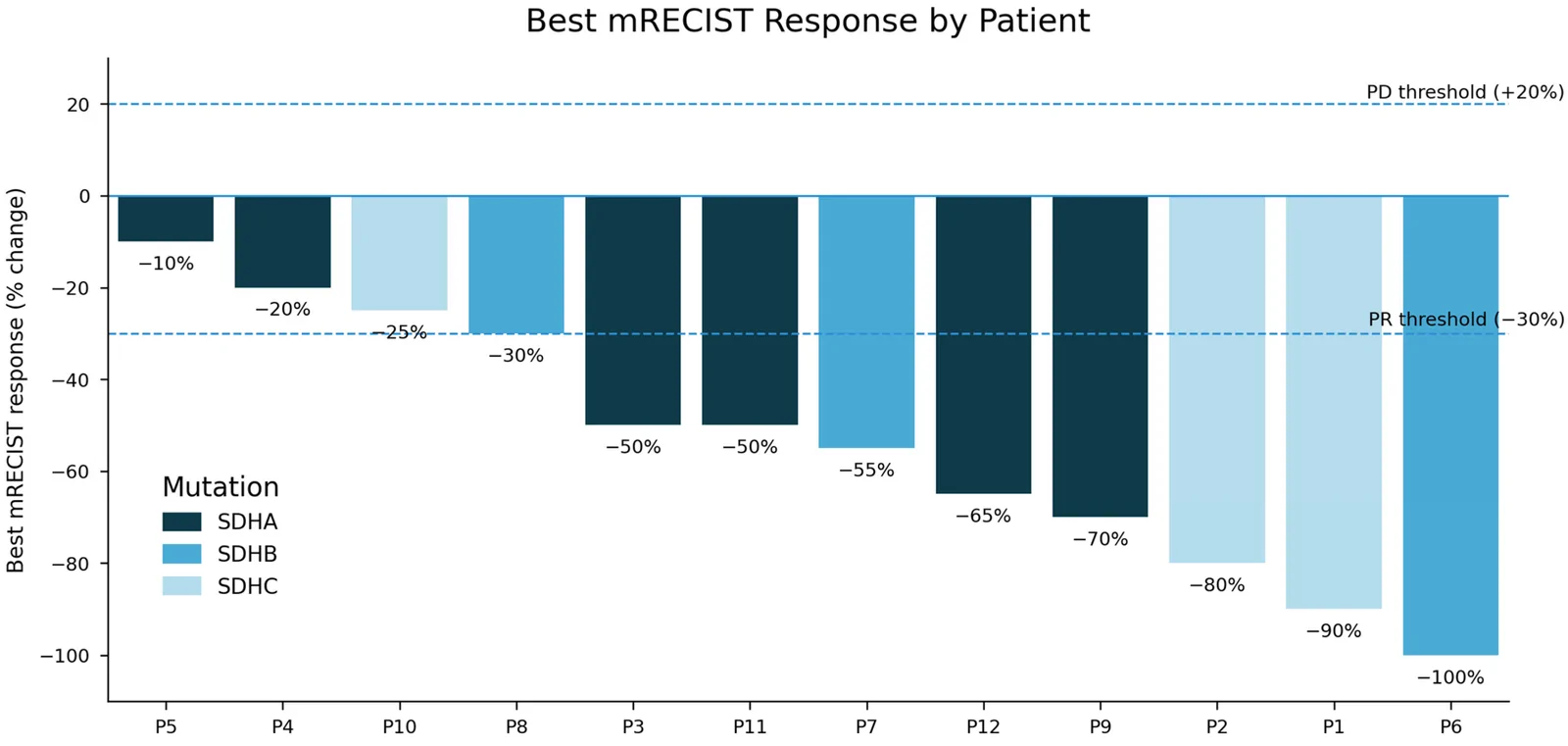
Waterfall plot of depth of response to selective internal radiation therapy.

At a median follow-up of 32 months (range, 3–77 months), the disease control rate was 100%. One patient had a complete response (8.3%), seven had partial responses (58.3%), and four had stable disease (33.3%), resulting in an ORR of 66.7%. Median depth of response was 52.5%. Two patients experienced disease progression during follow-up at 10 and 40 months, respectively. One patient died during follow-up from progressive peritoneal metastases, with hepatic disease controlled at last assessment. Progression-free and overall survival are shown in Figure 2; depth of response is shown in Figure 3.

## 4. Discussion

In this series of 12 patients treated with Y-90 SIRT for progressive, unresectable SDH-deficient GIST hepatic metastases, the disease control rate was 100% and the objective response rate was 66.7%, including one complete response. Median depth of response was 52.5%. Intrahepatic control was durable, with only two patients progressing at a median follow-up of 32 months, and the single death during follow-up was attributable to peritoneal rather than hepatic disease. Most patients experienced typical postembolization symptoms, including fatigue, nausea, and loss of appetite. One patient had a grade 3 or higher toxicity, with cholecystitis requiring cholecystectomy, and no clinically recognized delayed hepatic toxicity was documented.

SIRT is functionally high-dose transarterial brachytherapy with beta-emitting Y-90 particles. It delivers high doses of radiation through preferential arterial supply to tumors relative to background liver, leading to tumoral DNA damage and cellular death. It differs from bland or chemoembolization in that its principal effect is radiation rather than ischemia or regional chemotherapy, which is relevant for a tumor with poor sensitivity to conventional cytotoxic agents. Randomized data support SIRT in colorectal liver metastases, and NCCN guidelines have included radioembolization among liver-directed treatment options for selected hepatobiliary and neuroendocrine malignancies [5,6,7].

Activity was prescribed using MIRD, partition, or modified BSA methods according to device and institutional practice rather than to a common dosimetric target, so the median depth of response of 52.5% was achieved without a prespecified dose target. Device selection and dosimetry were determined by operator experience, institutional practice, and payer coverage rather than predefined clinical criteria; no statistically significant differences in response were observed between microsphere types. Tumor absorbed-dose thresholds predictive of response have been defined for hepatocellular carcinoma and colorectal liver metastases but not for GIST, and the dose required for response in SDH-deficient disease remains unknown; a prespecified dosimetric and response framework should therefore be captured prospectively in any future registry.

The depth and durability of response seen here exceed what has been reported for systemic agents studied in this subtype. Within the present cohort, response outcomes were also similar between treatment-naive patients and those previously exposed to systemic therapy. Unlike KIT/PDGFRA-mutated GIST, which is driven by gain-of-function signaling, SDH-deficient GIST is characterized by loss of SDH-complex activity, with impaired succinate metabolism causing succinate accumulation and inhibition of alpha-ketoglutarate-dependent enzymes [8]. An activated kinase can often be inhibited directly, whereas restoring a lost metabolic complex is considerably more difficult, which may partly explain the limited efficacy of systemic therapies in this subtype [2,9].

Imatinib has limited activity because most SDH-deficient tumors lack the activating KIT or PDGFRA alteration it is designed to suppress. Later-line TKIs may still exert antiangiogenic effects through VEGFR and related targets. In a small series of imatinib-resistant KIT/PDGFRA wild-type GIST, sunitinib produced one partial response and stable disease in five patients [10]. Regorafenib has also shown activity after prior TKI failure, with limited data suggesting activity in SDH-deficient disease [9,11]. Responses of the magnitude seen in this study have not been reported with either agent.

Prospective studies of other biologically plausible targets have also shown limited efficacy. Vandetanib produced no objective responses among nine children and adults with metastatic SDH-deficient GIST, although two patients had prolonged stable disease [12]. Linsitinib showed no objective responses in 20 patients, with a 9-month clinical benefit rate of 40% [13]. A phase Ib study of imatinib plus binimetinib enrolled six patients with SDH-deficient disease and showed an early efficacy signal, but the cohort was too small to define benefit [14]. Together, these studies have not established a consistently effective systemic option.

SDH loss also causes broad DNA hypermethylation, and MGMT promoter methylation has been identified preferentially in a subset of SDH-deficient GISTs [15]. Reduced MGMT-mediated DNA repair could increase sensitivity to temozolomide and potentially ionizing radiation, though this has not been tested directly. Earlier temozolomide studies in molecularly unselected GIST showed minimal activity [16], whereas preclinical models and small SDH-deficient series suggested greater sensitivity [8,9]. A phase II study (NCT03556384) was designed to evaluate this hypothesis, but all results remain pending. Guadecitabine likewise did not establish meaningful objective activity in a small multi-tumor study [17]. More recently, olverembatinib produced confirmed partial responses in six of 26 evaluable patients with TKI-failed SDH-deficient GIST, with an objective response rate of 23.1% [18]. No single systemic agent has produced consistent, durable disease control in this population [19].

Experience with liver-directed therapy in molecularly heterogeneous GIST provides useful context for these results. The liver and peritoneum are the principal sites of metastatic spread in GIST overall, and hepatic progression can become the dominant clinical problem even when extrahepatic disease remains controlled [20,21]. Kobayashi et al. evaluated hepatic artery chemoembolization in 110 patients, of whom 85 were radiologically evaluable; partial response occurred in 14%, stable disease in 74%, and progressive disease in 12%, with a median liver progression-free survival of 8.2 months and median overall survival of 17.2 months [3]. The initial GIST-specific SIRT experience was reported by Rathmann et al., who evaluated nine patients with progressive hepatic metastases during TKI therapy; three achieved a complete response, five a partial response, and one stable disease, with a median hepatic progression-free interval of 15.9 months and median overall survival of 29.8 months. No patient developed radiation-induced liver disease, although one required surgery for a persistent gastric ulcer [22]. More recently, Hohenberger et al. reported 26 patients with biopsy-proven GIST liver metastases and RECIST-documented progression, 24 of whom had received as many as four prior systemic treatment lines. Median hepatic progression-free survival was 16 months and median overall survival after SIRT was 28 months, with activity observed across KIT-mutant, PDGFRA-mutant, and quadruple wild-type disease and the longest median hepatic progression-free survival in the quadruple wild-type subgroup [4]. That series included two patients with SDH-deficient GIST. The response rate and durability in the present, genotype-restricted cohort compare favorably with these mixed-genotype series, which raises the possibility that SDH-deficient tumors are among the more radiosensitive GIST subtypes.

Locoregional treatment of GIST liver metastases is not limited to radioembolization. Surgical resection remains preferred when metastases are limited in number and anatomically favorable, and a recent meta-analysis confirmed a survival benefit for hepatectomy in the TKI era, although most patients with SDH-deficient GIST present with diffuse or bilobar disease that precludes complete resection [23]. Radiofrequency ablation offers a nonoperative alternative for a limited number of small, favorably located tumors, though its utility likewise diminishes as tumor number and size increase [24,25]. External beam radiotherapy has a more limited evidence base in GIST, reflecting the historical assumption of radioresistance that more recent series have begun to challenge [26]. Contemporary reviews emphasize that no single modality applies to all patients and that treatment selection should be individualized according to disease distribution, prior therapy, and resectability [27]. Radioembolization is well suited to the multifocal, bilobar pattern typical of SDH-deficient GIST because, unlike resection or ablation, it can address the entire liver in a single or staged session without requiring discrete, countable targets.

When extrahepatic disease is absent, stable, or comparatively indolent, hepatic control with SIRT could delay or prevent liver failure from becoming the dominant clinical event and may permit continuation of an effective systemic regimen or delay transition to a less effective line of therapy. Patients with substantial extrahepatic progression, by contrast, still require systemic management, as illustrated by the single death in this cohort. Temozolomide is also a radiosensitizer and has been combined with radioembolization in neuroendocrine tumors [28], making combination studies an additional research direction in a subtype for which both MGMT methylation and apparent radiosensitivity have now been described.

The median age in this cohort was 27 years, all patients carried a germline SDH variant, and expected survival is measured in years rather than months, so decisions about hepatic radiation are made against a much longer horizon than in colorectal or hepatocellular disease. Repeat treatment is a realistic expectation, as illustrated by the patient here who underwent a segmental infusion 2.5 years after an initial lobar treatment, and cumulative hepatic dose and preservation of functional liver reserve therefore become relevant to sequencing.

Delayed hepatic injury is the most direct of these concerns, with neuroendocrine tumors being the closest available analogue because those patients also survive long enough for it to become apparent. Radioembolization-induced chronic hepatotoxicity (RECHT) refers to clinical, laboratory, or imaging evidence of chronic liver disease arising at least six months after treatment in the absence of intrahepatic tumor progression. Currie et al. reviewed 98 patients who survived at least one year after radioembolization, of whom 54% had neuroendocrine tumors, and identified RECHT in 13%, with five deaths from hepatic decompensation; higher intrahepatic tumor volume and pre-existing cirrhosis were associated with its development [29]. No clinically recognized delayed hepatic toxicity was documented in this cohort.

This study is limited by its small sample size and retrospective design. The cohort was highly selected and lacked a comparator group, limiting inference regarding efficacy relative to systemic therapy or other liver-directed approaches. Enrollment across three centers introduced treatment heterogeneity in dosimetry methods and microsphere devices, which were not randomized; device selection reflected operator preference, institutional practice, and payer coverage. Imaging response was assessed by the treating investigators rather than through independent, blinded central review, and mRECIST is not formally validated for GIST. Follow-up duration also varied considerably among patients (range, 3–77 months), which may reduce precision and potentially overstate the apparent durability of disease control. Given the rarity of SDH-deficient GIST, a randomized trial is unlikely to be feasible. A large multicenter or national registry would therefore be valuable to further define the role of SIRT for SDH-deficient GIST, particularly given these promising initial results.

## 5. Conclusions

Y-90 SIRT produced durable intrahepatic disease control with limited serious toxicity in this multicenter cohort of patients with progressive, unresectable SDH-deficient GIST liver metastases, with an objective response rate that exceeds what has been reported for available systemic agents in this subtype. These results suggest that SDH-deficient GIST may be more radiosensitive than the historical assumption of radioresistance in GIST would predict. SIRT is a reasonable option to consider in carefully selected patients with liver-dominant progression, and a multicenter registry is warranted to define patient selection, dosimetric targets, and long-term outcomes.

## Figures and Tables

**Table 1:** Summary of demographic data, procedural details, and outcomes.

Demographic Data	
Median age, years (range)	27 (17–57)
Gender	8 female/4 male
Mutational status, N (%)	
SDHA	6 (50%)
SDHB	3 (25%)
SDHC	3 (25%)
Prior surgery	9 (75%)
Prior systemic therapy	7 (58.3%)
SIRT Procedural Data	
Microspheres, N (%)	
Glass	5 (41.7%)
Resin	6 (50%)
Both glass and resin	1 (8.3%)
Infused volume, N (%)	
Bilobar	9 (75%)
Unilobar	1 (8.3%)
Segmental	1 (8.3%)
Combination	1 (8.3%)
Imaging Outcomes	
Best mRECIST response, N (%)	
Stable disease	4 (33.3%)
Partial response	7 (58.3%)
Complete response	1 (8.3%)
Objective response rate	8 (66.7%)
Disease control rate	12 (100%)
Median depth of response	52.5%
Median follow-up, months (range)	32 (3–77)
Progression during follow-up	2 (16.7%)
Death during follow-up	1 (8.3%)

## Data Availability

The data supporting the findings of this study are available from the corresponding author upon reasonable request and subject to institutional and privacy restrictions.

## Abbreviations

The following abbreviations are used in this manuscript:

BSA	Body surface area
CTCAE	Common Terminology Criteria for Adverse Events
GIST	Gastrointestinal stromal tumor
HIF	Hypoxia-inducible factor
MIRD	Medical Internal Radiation Dose
mRECIST	Modified Response Evaluation Criteria in Solid Tumors
NCCN	National Comprehensive Cancer Network
ORR	Objective response rate
PDGFRA	Platelet-derived growth factor receptor alpha
PFS	Progression-free survival
RECHT	Radioembolization-induced chronic hepatotoxicity
SDH	Succinate dehydrogenase
SIRT	Selective internal radiation therapy
Y-90	Yttrium-90
